# Three-year results of a prospective, controlled clinical study evaluating treatment of intra-bony defects with PRF or EMD- a randomized control trial

**DOI:** 10.1186/s12903-026-08845-y

**Published:** 2026-06-15

**Authors:** Ferenc Dőri, Florina Németh, Nicole Arweiler, Eleonora Solyom

**Affiliations:** 1 Department of Periodontology, Semmelweis University, Budapest, Hungary; 2 University of Marburg, Marburg, Germany

**Keywords:** Periodontitis, Intrabony defects, Enamel matrix derivative, Platelet-rich fibrin, Periodontal regeneration

## Abstract

**Background:**

Periodontal regenerative surgery is a well-established treatment modality for intrabony periodontal defects. Enamel matrix derivative (EMD) is one of the most extensively documented biologic agents, whereas platelet-rich fibrin (PRF) has emerged as a promising autologous alternative. This prospective randomized controlled clinical study aimed to compare the long-term clinical outcomes of PRF and EMD in the treatment of periodontal intrabony defects.

**Methods:**

Thirty patients with periodontitis Stage III/IV, grade B/C and at least one intrabony defect [probing pocket depth (PPD) ≥ 6 mm; intrabony component ≥ 4 mm] were randomly assigned to surgery using PRF (test group) or EMD (control group). The primary outcome was clinical attachment level (CAL) gain. Secondary outcomes included PPD reduction, gingival recession (GR), and bone sounding (BS) changes. Clinical parameters were recorded at baseline, 6 months, and 3 years postoperatively. Intergroup comparisons of mean clinical changes were performed using appropriate parametric and nonparametric statistical tests (α = 0.05).

**Results:**

Twenty-six patients completed the 3-year follow-up. Both treatment modalities resulted in significant clinical improvements that remained stable over time. At 6 months, mean PPD reduction was 4.40 ± 2.44 mm in the PRF group and 3.53 ± 2.67 mm in the EMD group, while mean CAL gain measured 3.87 ± 2.20 mm and 2.13 ± 2.80 mm, respectively. GR increase was lower in the PRF group compared with the EMD group (0.53 ± 1.13 mm vs. 1.40 ± 1.45 mm), although the difference did not reach statistical significance (*p* = 0.079). At 3 years, mean PPD reduction was 3.69 ± 1.70 mm in the PRF group and 3.31 ± 3.45 mm in the EMD group, while CAL gain measured 3.77 ± 1.48 mm and 2.54 ± 3.62 mm, respectively. A significantly lower increase in GR was observed in the PRF group compared with the EMD group at 3 years (− 0.08 ± 0.64 mm vs. 0.77 ± 1.30 mm; *p* = 0.046). No statistically significant intergroup differences were detected for PPD reduction, CAL gain, or BS reduction.

**Conclusions:**

PRF and EMD provided comparable and stable long-term clinical improvements following regenerative periodontal surgery of intrabony defects. PRF may represent a reliable autologous alternative to EMD in periodontal regenerative therapy.

**Trial registration:**

ClinicalTrials.gov registration number NCT07183631 retrospectively registered, 14/09/2025.

## Background

Periodontal regenerative surgery is a well-established treatment approach for intrabony defects; however, achieving complete and fully predictable periodontal regeneration remains challenging. The European Federation of Periodontology (EFP) S3-level clinical practice guideline recommends regenerative periodontal surgery for intrabony defects ≥ 3 mm, based on substantial evidence demonstrating improved clinical attachment gain and probing depth reduction compared with open flap debridement alone [1]. Previous landmark clinical studies have demonstrated the long-term efficacy of regenerative approaches in the treatment of intrabony periodontal defects, including the use of enamel matrix derivative (EMD), bone grafts and combination therapies [2–4].

Among biologic agents, enamel matrix derivative (EMD) is one of the most extensively studied and most predictable material. Numerous in vitro studies have evaluated the effects of EMD on osteoblasts and periodontal fibroblasts. These studies indicate that EMD promotes the proliferation of PDL fibroblasts, enhances their protein synthesis, and supports the formation of mineralized nodules. While specific signaling molecules such as IGF-1, IGF-2, PDGF-BB, TNF, TGF-β, and IL-1β have not been directly identified within EMD, the extract appears to foster mesenchymal cell proliferation by inhibiting epithelial growth and encouraging autocrine growth factor release from periodontal fibroblasts [5, 6].

A promising strategy to support periodontal regeneration is the use of platelet concentrates, particularly platelet-rich fibrin (PRF). Nearly 40 randomized clinical trials have investigated its regenerative potential, showing PRF to be particularly effective in soft tissue healing, though its benefits in hard tissue regeneration remain a topic of ongoing debate [7–13].

PRF is known to contain several key growth factors, including platelet-derived growth factor (PDGF), transforming growth factor-beta (TGF-β), and insulin-like growth factors (IGFs). TGF-β plays a dual role: it stimulates the production of extracellular matrix proteins such as collagen and fibronectin by osteoblasts and fibroblasts, while also exerting anti-inflammatory effects [13]. PDGF regulates mesenchymal cell proliferation, migration, and survival, whereas IGFs help prevent apoptosis and promote cell proliferation and differentiation [14]. In addition to growth factors, PRF also contains both proinflammatory cytokines, such as tumor necrosis factor-alpha (TNF-α), interleukin-1 beta (IL-1β), and interleukin-6 (IL-6), as well as anti-inflammatory cytokines like interleukin-4 (IL-4) and vascular endothelial growth factor (VEGF) [9, 11, 15, 16]. Experimental studies support PRF’s regenerative capabilities. Li et al. demonstrated that PRF enhanced the migration and proliferation of periodontal progenitor cells in vitro, producing effects comparable to those of osteogenic media and superior to platelet-poor plasma [17]. In clinical settings, PRF application has been associated with bone fill in peri-implant defects. Chang et al. proposed that PRF promotes healing of intrabony periodontal defects by upregulating signaling molecules such as phosphorylated extracellular signal-regulated kinase (p-ERK), osteoprotegerin (OPG), and alkaline phosphatase (ALP) in regenerating tissues [18].

Although both enamel matrix derivative (EMD) and platelet-rich fibrin (PRF) have demonstrated favorable clinical outcomes in periodontal regenerative therapy, limited evidence is available regarding their comparative long-term efficacy in the treatment of intrabony periodontal defects. In particular, data directly comparing the stability of clinical outcomes over extended follow-up periods remain scarce. Therefore, further randomized clinical studies are needed to clarify whether PRF may represent a reliable alternative to EMD in periodontal regeneration.

The aim of the present randomized controlled clinical study was to compare the 3-year clinical outcomes of PRF and EMD in the surgical treatment of periodontal intrabony defects.

The null hypothesis of the study was that no statistically significant differences would be observed between PRF and EMD in terms of clinical attachment level gain, probing pocket depth reduction, gingival recession, and bone sounding changes after periodontal regenerative surgery.

## Methods

The study protocol follows the recommendations of the CONSORT guidelines [19]. Registration number: NCT07183631.

### Study design and patient selection

This prospective randomized controlled clinical study was conducted at the Department of Periodontology, Semmelweis University, Budapest, Hungary. Patient recruitment and surgical treatment were performed between [09. 2017.] and [10.2018.], while the final 3-year follow-up examinations were completed in [10.2021.]. During the early postoperative period, patients were examined at 1 month, 3 months, and 6 months. Thereafter, follow-up visits were scheduled at 6-month intervals.

**Fig. 1:**
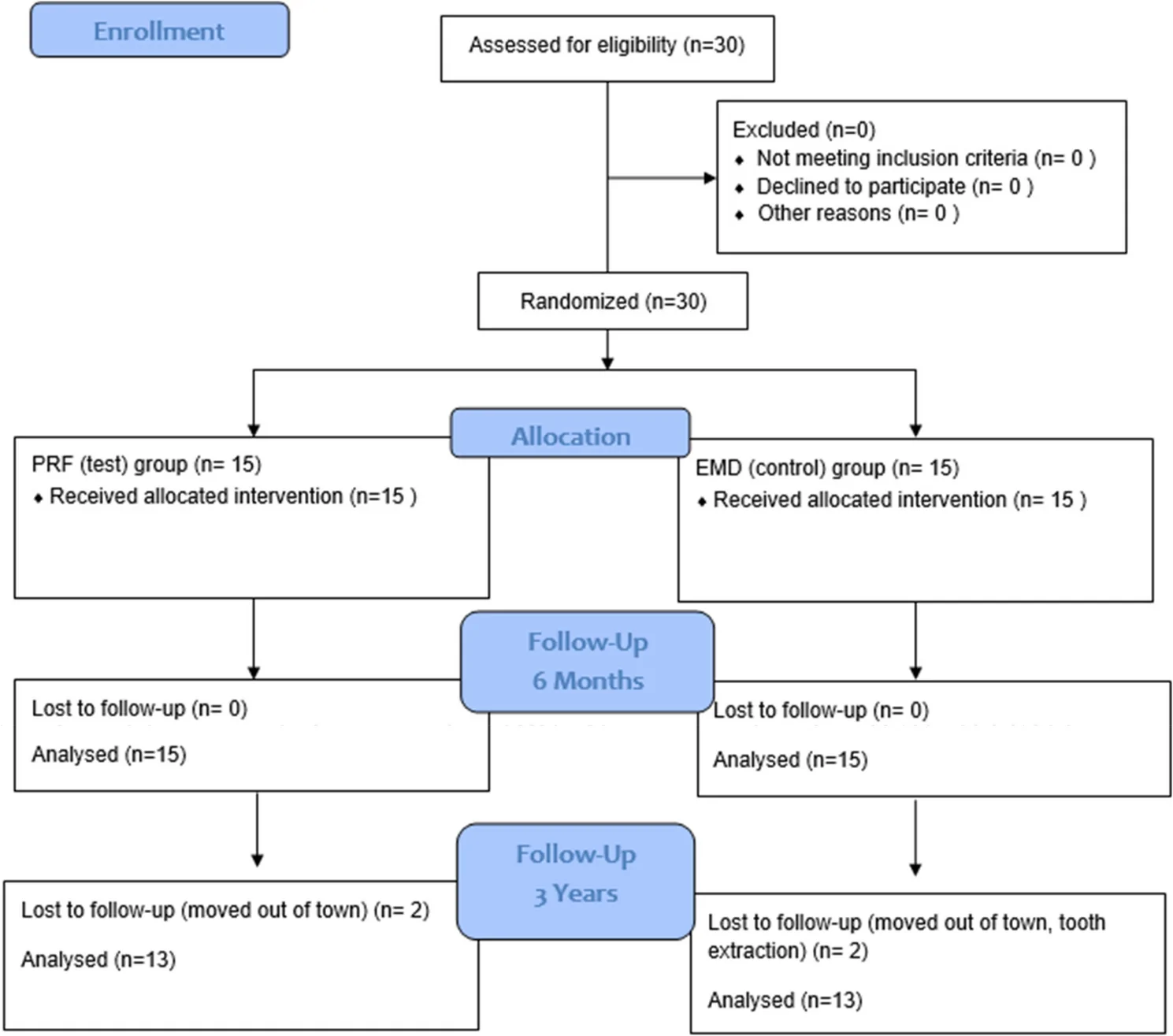
Patients follow up according to CONSORT flow-diagram

A total of 30 patients (18 females and 12 males) diagnosed with Stage III/IV, Grade B periodontitis were included in this randomized parallel-design study, with 15 patients assigned to each group after providing informed consent. First evaluation was performed 6 months after surgery. The study adhered to the ethical principles outlined in the Helsinki Declaration of 1983 and 2013 [20]. However, only 26 patients (15 females and 11 males) completed the three-year evaluation. Four patients were lost to follow-up due to one participant tooth had to be removed and three relocating (Fig. 1).

Prior to enrolment, all patients received thorough oral hygiene instructions, as well as full-mouth supra- and subgingival treatment, scaling and root planing using ultrasonic and hand instruments. Subgingival scaling was conducted under local anaesthesia. Three months post-initial therapy, periodontal status was controlled to determine eligibility based on the following inclusion criteria:

1. No systemic conditions affecting treatment outcomes.
2. Good oral hygiene [plaque index (PI) < 1; gingival index (GI) < 1] [21].
3. Compliance with the maintenance program.
4. Presence of one intra-bony defect with a probing pocket depth (PPD) of ≥ 6 mm and an intra-bony component of ≥ 4 mm confirmed by radiographs.

Exclusion criteria were the following: (1) significant medical conditions: irradiation in the maxillofacial area, uncontrolled diabetes or hypertension, systemic steroid or bisphosphonate treatment, pregnancy, or lactation, infectious diseases, (2) age < 18 years, (3) smokers (> 5 cigarettes/day), (4) high residual inflammation (FMBS > 25%), (5) poor oral hygiene (FMPS > 25%), and (6) defect-related factors: furcation involvement, endo-periodontal defects or multi-tooth defects.

### Clinical assessment

At study-designated teeth, the following clinical parameters were assessed one week before, at six months, and 3 years post-surgery using a standardized periodontal probe (PCP 12, Hu-Friedy, Chicago, IL, USA):

- Plaque index (PI) [21].
- Gingival index (GI) [21].
- Probing pocket depth (PPD).
- Gingival recession (GR).
- Clinical attachment loss (CAL).
- Bone Sounding (BS).

The primary outcome was the CAL. Measurements were recorded at six sites per tooth: mesiovestibular (mv), midvestibular (v), distovestibular (dv), mesiooral (mo), midoral (o), and distooral (do) by a calibrated investigator blinded to the surgical procedure (FN). The cemento-enamel junction (CEJ) was used as the reference point. When the CEJ was not visible, a restoration margin was used. The study reports only measurements at the same, at baseline the deepest point of the selected defects. Baseline and postoperative radiographs were taken using the long-cone paralleling technique.

### Intra-examiner reproducibility

To ensure measurement consistency, the examiner was calibrated using five patients, each with ten teeth exhibiting PPD > 6 mm on at least one aspect. Patients were evaluated twice, 48 h apart, and calibration was accepted if baseline and follow-up measurements matched to the millimeter in at least 90% of cases. Intra-examiner reproducibility was assessed using the intraclass correlation coefficient (ICC). Repeated measurements performed 48 h apart demonstrated excellent reproducibility, with ICC values exceeding 0.90 for all clinical parameters. The examiner remained blinded to the surgical procedures.

### Randomization procedure

Patients were randomly assigned in a 1:1 ratio to either the test group (PRF) or the control group (EMD). Group assignments were concealed in sequentially numbered, opaque, sealed envelopes, which were opened only at the time of surgery. This procedure ensured that neither the patient nor the investigators were aware of the allocation.

### Masking

The clinical examiner responsible for outcome assessment was blinded to the treatment allocation throughout the study period. Due to the nature of the surgical procedures, the operating surgeon could not be blinded to the assigned treatment. Patients were unaware of group allocation until the time of surgery.

### Statistical analysis

Data were expressed as mean ± standard deviation (SD). Normality of distribution was assessed using the Shapiro–Wilk test. Intragroup comparisons between baseline and follow-up examinations (6 months and 3 years) were performed using paired Student’s t-tests for normally distributed variables and Wilcoxon signed-rank tests for non-normally distributed variables. Intergroup comparisons between the PRF and EMD groups were performed using independent Student’s t-tests or Mann–Whitney U tests, as appropriate.

Changes from baseline (Δ values) for probing pocket depth (PPD), gingival recession (GR), clinical attachment level (CAL), and bone sounding (BS) were calculated and compared between groups. A p-value < 0.05 was considered statistically significant. Statistical analyses were performed using SPSS software (version XX; IBM Corp., Armonk, NY, USA).

### Sample size calculation

The primary outcome variable for sample size estimation was clinical attachment level (CAL) gain from baseline to 6 months. Based on previous clinical studies evaluating regenerative periodontal treatment of intrabony defects, a standard deviation of approximately 2.0 mm for CAL gain was assumed. With 15 participants per group, a two-sided significance level of α = 0.05 provides 80% power to detect a clinically relevant between-group difference of 2.0 mm in CAL. This sample size was considered appropriate given the feasibility of patient recruitment and the planned longer follow-up.

### Surgical procedures

All surgeries were performed under local anesthesia by the same surgeon (F.D.). The surgical treatment involved intracrevicular incisions, raising full-thickness flaps vestibularly and orally without vertical releasing incisions, debridement of granulation tissue, scaling and root planing using hand and ultrasonic instruments. Papilla preservation principles were applied whenever defect anatomy allowed preservation of the interdental soft tissues; however, a strictly standardized MIST or modified MIST protocol was not used.

### Test group (PRF application)

Platelet-rich fibrin (PRF) was prepared according to the manufacturer’s instructions. A blood sample was collected from the cubital vein in 10 ml tubes without anticoagulant and centrifuged at 3000 rpm for 10 min using a GLO GT416 table centrifuge *(GLO Glotech Co. Ltd*,* Korea)* and GLO PRP collection kit *(GLO PRP-Kit*,* Glotech Co*,* Asan Choong-nam*,* Korea/ Glofinn*,* Finland*). Without anticoagulant, platelet activation occurred rapidly, leading to coagulation cascade initiation. The centrifugation process resulted in three layers:

- Platelet-poor plasma (PPP) (top layer).
- Fibrin clot (PRF) (middle layer).
- Red blood cells (RBC) (bottom layer).

**Fig. 2:**
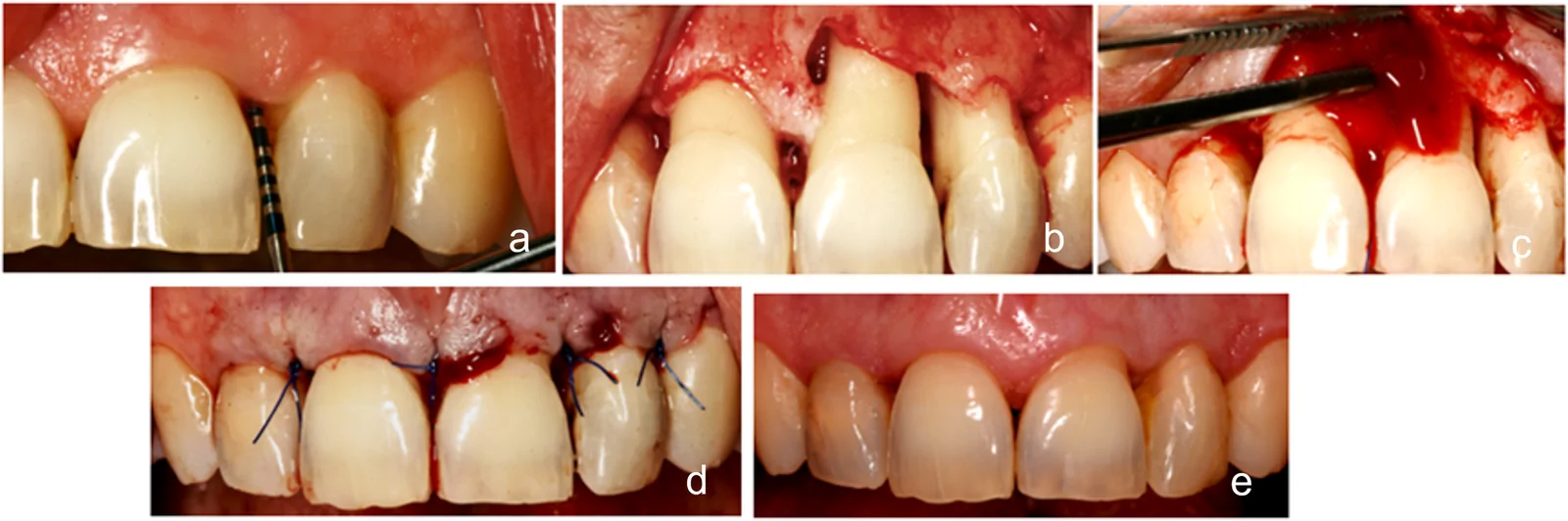
Application of PRF to the periodontal bony defects. PRF case: **a** preoperative measurements; **b** cleaned periodontal defect; **c** insertion of the PRF clot; **d** sutures; **e** clinical picture after three years

The fibrin clot (PRF) was extracted and applied directly to the defect in its gel form (Fig. 2).

### Control group (EMD application)

**Fig. 3:**
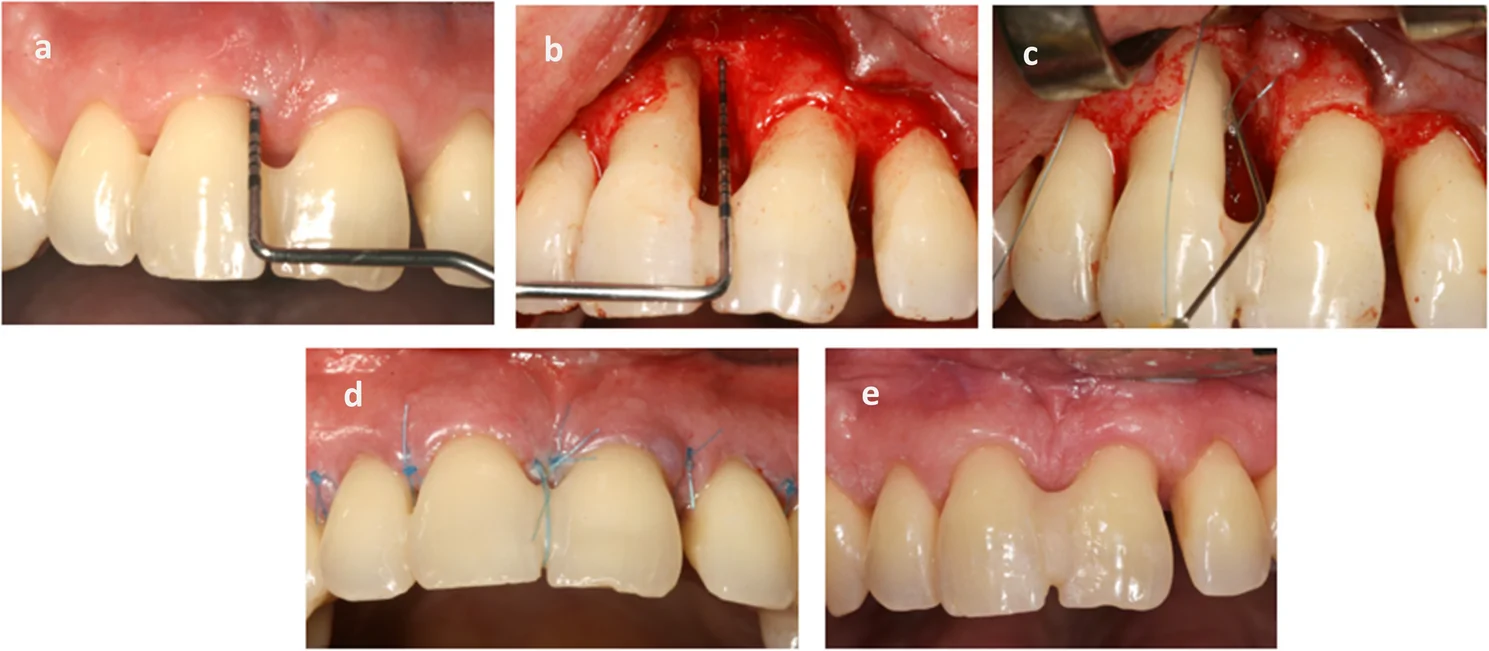
Application of EMD to the periodontal bony defects. **a** preoperative measurements; **b** cleaned periodontal defect; **c** EMD application on the conditioned and dried root surface; **d** sutures; **e** clinical picture after three y

In the control group, the root surface was conditioned with 24% ethylenediaminetetraacetic acid (EDTA) gel for two minutes, washed with physiological saline solution, dried, and treated with enamel matrix derivative (EMD), (“*Emdogain”*,* Straumann*,* Switzerland)* per manufacturer recommendations. The flap was then closed with vertical mattress sutures (Fig. 3).

### Postoperative care

All patients received systemic antibiotics (Amoxicillin 1000 mg, twice daily) for one week following surgery. Post-surgical care consisted of 0.2% chlorhexidine rinses twice daily for two weeks and analgesics (Ibuprofen 600 mg, twice daily for three days). Sutures were removed after 14 days.

Patients were reviewed monthly during the first six months and at three-month intervals thereafter. During the first six months, no probing or subgingival instrumentation was performed. Recall visits focused on reinforcement of oral hygiene and professional supragingival cleaning. Clinical evaluations were carried out at 6 months and again at the 3-year follow-up.

## Results

Postoperative healing was uneventful. No adverse reactions to antibiotics or analgesics were reported. Patients in both groups expressed high satisfaction with treatment, largely due to the possibility to tooth retention. Four patients were lost to follow-up due to tooth extraction in one patient and relocation in three patients (Fig. 1).

Baseline patient characteristics, defect distribution and characteristics are presented in Table 1, with no statistically significant differences found between groups.

**Table 1:** Baseline patient, tooth and defect characteristics

Variable	PRF group (*n* = 15)	EMD group (*n* = 15)
Age (years, mean ± SD)	42.2 ± 8.3	45.7 ± 7.9
Gender (M/F)	7 / 8	5 / 10
Smokers	*0*	*0*
Tooth type	*F: 7 / PM: 11 / M: 12*	
Splinting	*Yes: 12 / No: 18*	
Type of bony defects	*1-wall: 8 / 2-wall: 16 / 3-wall: 6*	

Baseline clinical parameters are presented in Table 2. No statistically significant differences were observed between the PRF and EMD groups for any evaluated parameter at baseline (*p* > 0.05), indicating comparable clinical conditions before treatment.

**Table 2:** Baseline clinical parameters

Parameter	PRF group (mean ± SD)	EMD group (mean ± SD)	*p*-value
PPD (mm)	*8.93 ± 1.91*	*8.27 ± 2.40*	*0.407*
GR (mm)	*1.27 ± 1.58*	*1.27 ± 1.22*	*1.000*
CAL (mm)	*10.20 ± 2.24*	*9.53 ± 2.39*	*0.437*
BS (mm)	*10.60 ± 1.68*	*10.14 ± 2.25*	*0.539*

### Test group

**Fig. 4:**
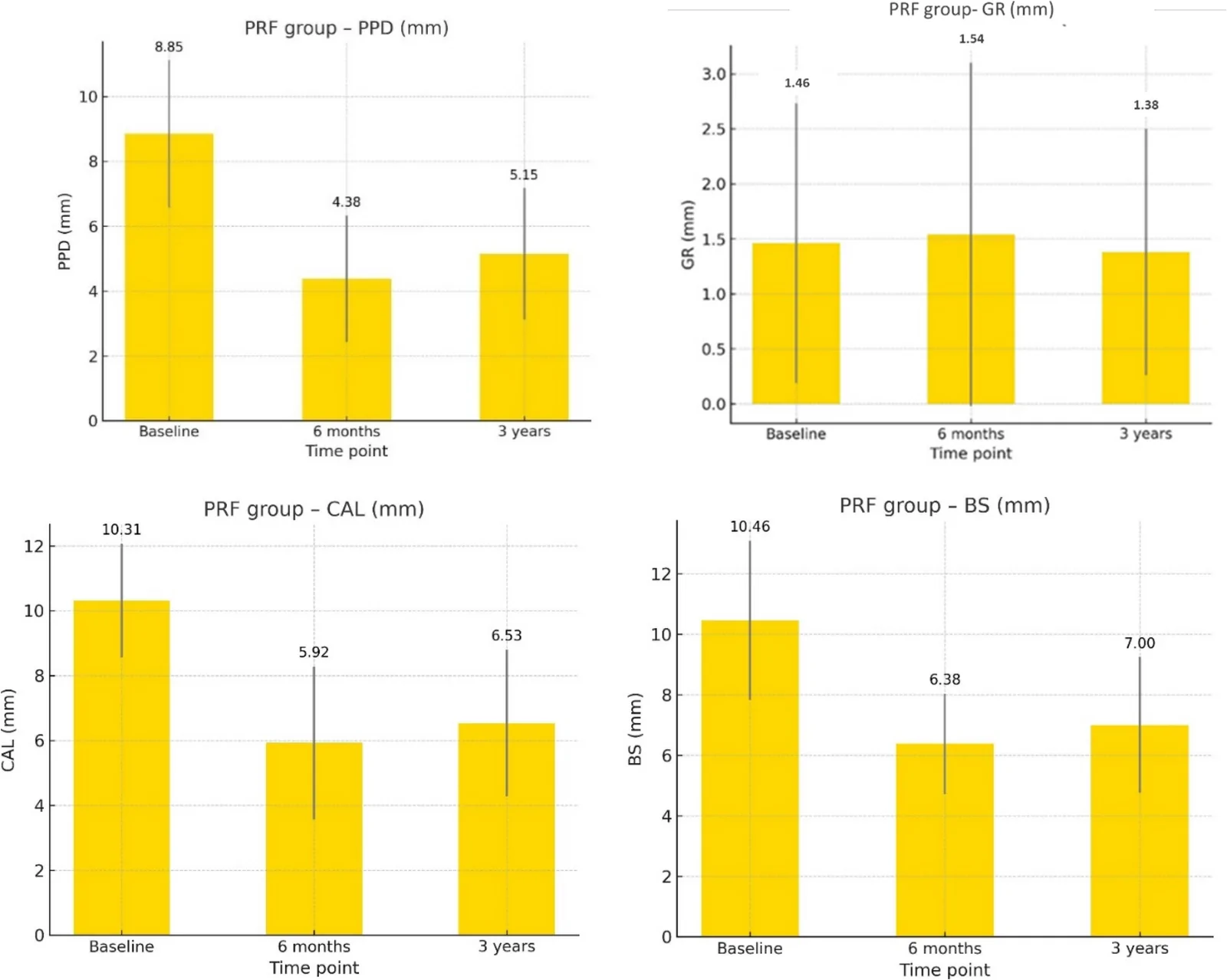
PRF (Test) group: PPD-, GR-, CAL-, BS-values, preoperative, at 6 months, and 3 yearspostoperative

At 6 months after therapy, the test group showed a reduction in mean PPD from 8.85 ± 2.27 mm to 4.38 ± 1.95 mm (*p* < 0.001) and a change in mean CAL from 10.31 ± 1.75 to 5.92 ± 2.36 mm (*p* < 0.001). At three years, mean PPD and CAL measured 5.15 ± 2.03 and 6.53 ± 2.26, respectively. At three years, both PPD and CAL were statistically significantly improved compared with baseline (*p* < 0.001) without statistically significant differences between the 6 months and three-year results (Fig. 4).

After 6 months, the test group showed an increase in mean GR from 1.46 ± 1.27 to 1.54 ± 1.56 mm (*p* < 0.989). At three years, mean GR measured 1.38 ± 1.12 mm without statistically significant differences between the 6 months and three-year results (*p* < 0.957). After 6 months, the test group showed significant reduction in mean BS from 10.46 ± 2.63 to 6.38 ± 1.66 mm. An elevated BS was detected after three years 7.00 ± 2.24 mm (*p* < 0.001) (Fig. 4).

**Fig. 5:**
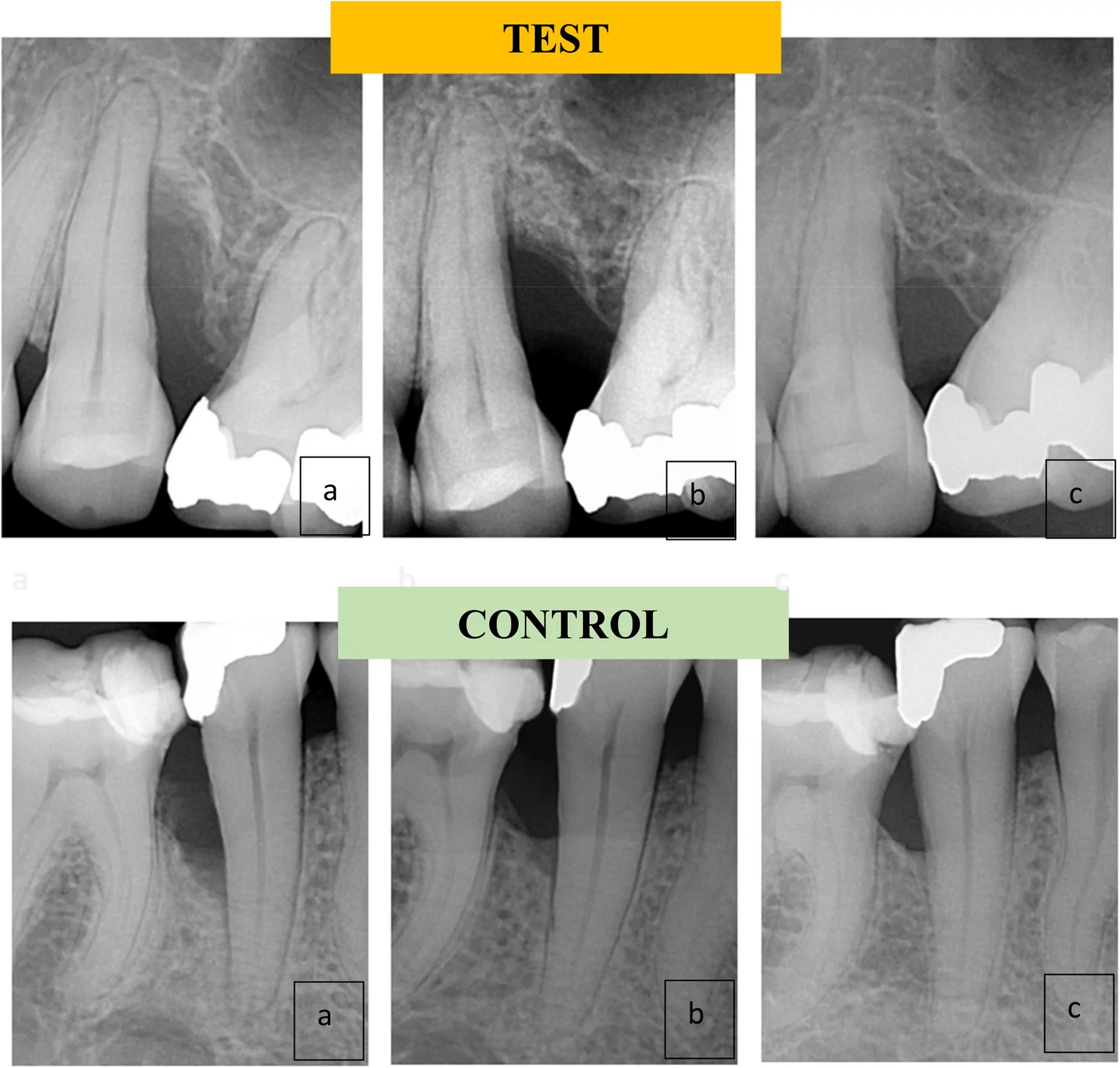
Representative radiographic images of PRF- and EMD-treated intrabony defects obtained preoperatively, at 6 months, and at the 3-year follow-up: (**a**) preop; (**b**) 6 months postoperatively; (**c**) three years postoperatively

Representative radiographic images are presented in Fig. 5 for illustrative purposes only. No quantitative radiographic assessment or standardized linear radiographic measurements were performed in the present study (Fig. 5).

### Control group

**Fig. 6:**
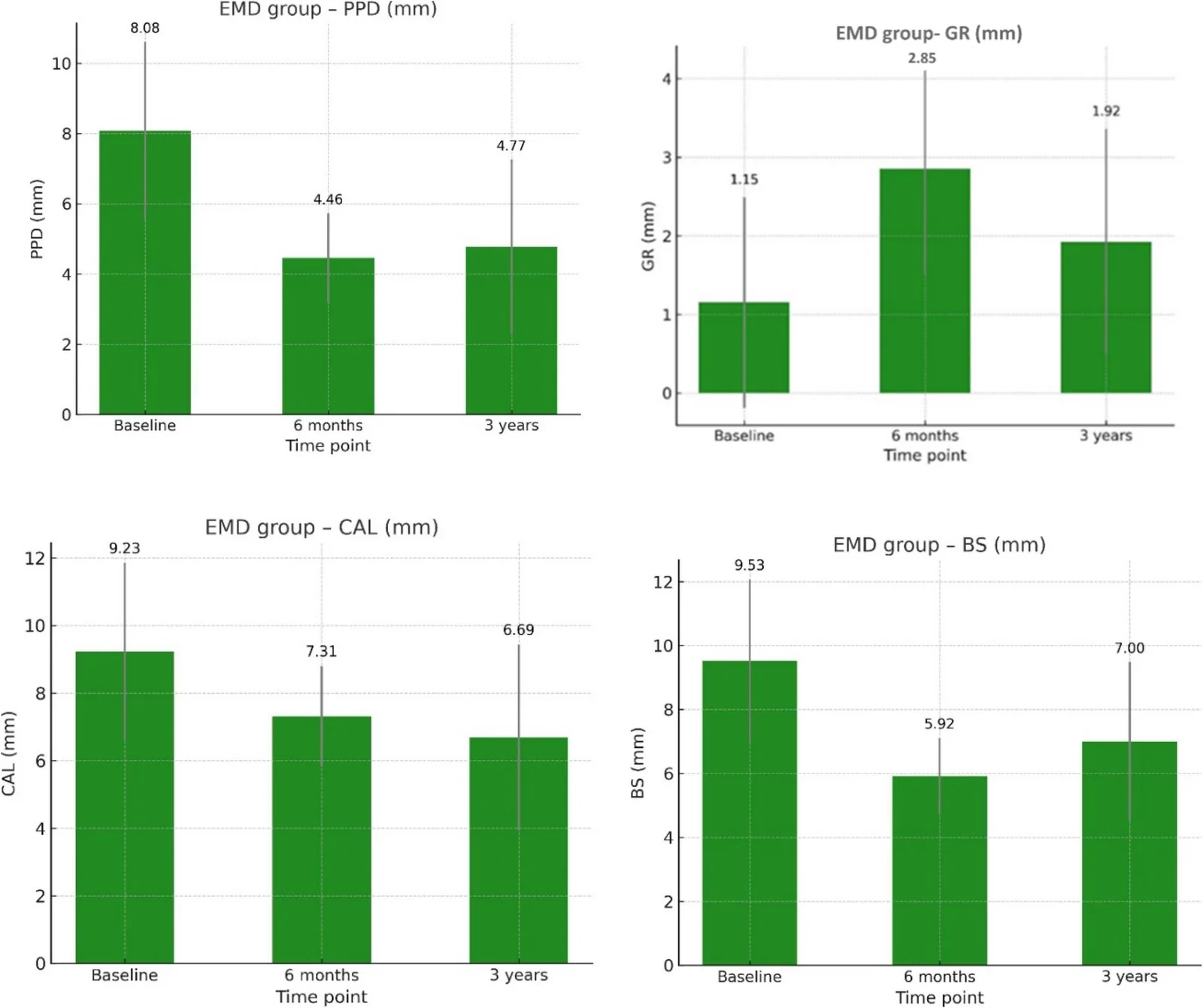
EMD (Control) group: PPD-, GR-, CAL-, BS-values, preoperative, at 6 months, and 3 yearspostoperative

In the control group, after 6 months, mean PPD was reduced from 8.08 ± 2.53 to 4.46 ± 1.27 mm (*p* < 0.001;) and mean CAL changed from 9.23 ± 2.62 to 7.31 ± 1.49 mm (*p* < 0.001). At three years, mean PPD and CAL measured 4.77 ± 2.49 and 6.69 ± 2.75 mm, respectively, and were still statistically significantly improved compared with baseline (Fig. 6).

After 6 months, the control group showed an increase in mean GR from 1.15 ± 1.34 to 2.85 ± 1.34 mm (*p* < 0.001). At three years, mean GR measured 1.92 ± 1.44 mm with not statistically significant differences between the 6 months and three-year results (*p* < 0.001). After 6 months, the control group showed significant reduction in mean BS from 9.53 ± 2.54 to 5.92 ± 1.19 mm. At three years an elevated BS measured 7.00 ± 2.48 mm (*p* < 0.001) (Fig. 6).

Although no radiographic evaluation was performed, a stable and well-defined bone fill was visible on the radiographs in all cases (Fig. 5).

### Inter-group analysis

**Fig. 7:**
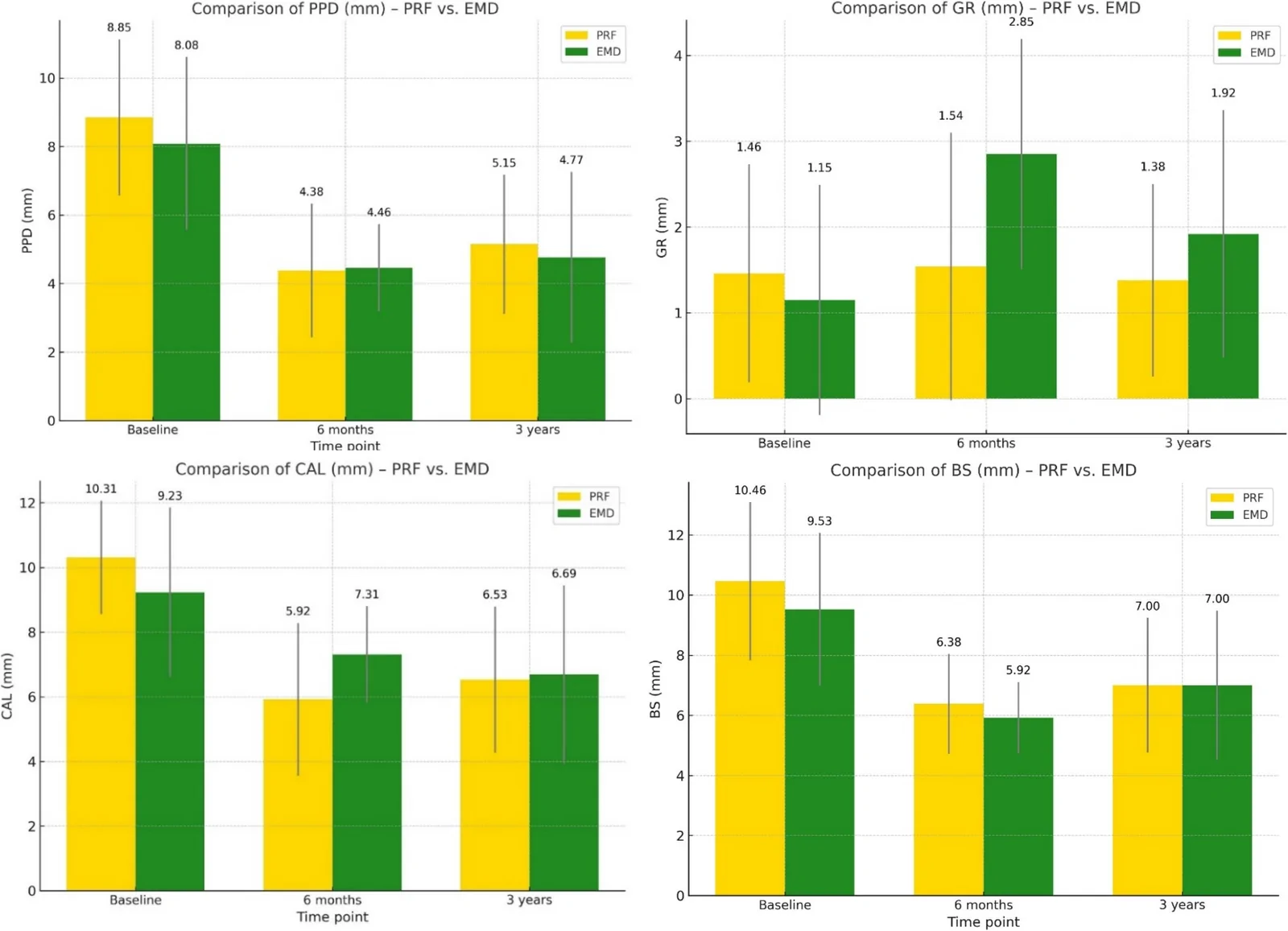
Inter-group PPD-, GR-, CAL-, BS-values; preoperative, 6 months, and 3 years differences between the test and the control group

The inter-group analysis confirmed comparable outcomes between the two treatment modalities. The test treatment, at 6 months, yielded statistically significantly lower GR than the control one (*p* < 0.031)(Fig. 7).

Both treatment modalities resulted in significant clinical improvements at 6 months (Table 3). Mean PPD reduction was 4.40 ± 2.44 mm in the PRF group and 3.53 ± 2.67 mm in the EMD group. Mean CAL gain measured 3.87 ± 2.20 mm and 2.13 ± 2.80 mm in the PRF and EMD groups, respectively. Mean GR increase was lower in the PRF group (0.53 ± 1.13 mm) compared with the EMD group (1.40 ± 1.45 mm), although the intergroup difference did not reach statistical significance (*p* = 0.079). No statistically significant intergroup differences were detected for any clinical parameter changes.

**Table 3:** Summarizes the mean clinical changes from baseline to 6 months in both treatment groups

Parameter	PRF mean change ± SD	EMD mean change ± SD	*p*-value
PPD reduction (mm)	4.40 ± 2.44	3.53 ± 2.67	0.362
GR increase (mm)	0.53 ± 1.13	1.40 ± 1.45	0.079
CAL gain (mm)	3.87 ± 2.20	2.13 ± 2.80	0.070
BS reduction (mm)	3.47 ± 2.33	3.87 ± 3.18	0.697

Table 4 summarizes the mean clinical changes from baseline to the 3-year follow-up. Both treatment modalities demonstrated stable long-term clinical improvements in all evaluated parameters. Mean PPD reduction was 3.69 ± 1.70 mm in the PRF group and 3.31 ± 3.45 mm in the EMD group, while mean CAL gain measured 3.77 ± 1.48 mm and 2.54 ± 3.62 mm, respectively. A significantly lower increase in gingival recession was observed in the PRF group compared with the EMD group at 3 years (− 0.08 ± 0.64 mm vs. 0.77 ± 1.30 mm; *p* = 0.046). No statistically significant intergroup differences were detected for PPD reduction, CAL gain, or BS reduction.

**Table 4:** Summarizes the mean clinical changes from baseline to three years in both treatment groups

Parameter	PRF mean change ± SD	EMD mean change ± SD	*p*-value
PPD reduction (mm)	3.69 ± 1.70	3.31 ± 3.45	0.722
GR increase (mm)	−0.08 ± 0.64	0.77 ± 1.30	0.046
CAL gain (mm)	3.77 ± 1.48	2.54 ± 3.62	0.268
BS reduction (mm)	3.46 ± 1.90	2.54 ± 3.57	0.419

## Discussion

Periodontal regeneration remains a central challenge in the treatment of advanced periodontitis due to the need for coordinated regeneration of multiple tissues, including cementum, periodontal ligament and alveolar bone. Biologic agents such as EMDs and platelet concentrates like PRF have emerged as promising strategies to enhance regenerative outcomes.

In this randomized clinical trial, in both PRF and EMD group a significant clinical improvement can be detected in the treatment of intrabony periodontal defects. Our findings are already evident at six months and remained stable over the three-year follow-up period. Probing pocket depth (PPD) and CAL improved markedly in both groups, with slightly greater early gains in the PRF group, although these differences diminished over time. This pattern mirrors previous reports indicating that PRF may provide an advantage during the initial healing phase, while long-term outcomes tend to converge with those of EMD [22].

At six months, significantly lower gingival recession was observed in the PRF group compared with the EMD group, suggesting a favorable soft tissue response associated with PRF. Interestingly, this tendency remained evident at the 3-year follow-up, where the PRF group demonstrated significantly lower GR increase compared with the EMD group. These findings may reflect the favorable soft tissue healing properties of PRF, potentially related to its fibrin matrix and gradual release of growth factors supporting angiogenesis and tissue maturation. Our findings are in line with Aydemir et al., who reported mean GR values of 2.08 ± 0.97 mm after six months in PRF-treated defects, comparable to our observations [22].

In terms of PPD and CAL, there were no statistical significant difference between test and control group at six months. At three years, these improvements were largely maintained, with final PPD values of 5.15 ± 2.03 mm (PRF) and 4.77 ± 2.49 mm (EMD), and CAL values of 6.53 ± 2.26 mm and 6.69 ± 2.75 mm, respectively. These outcomes closely parallel those of Iorio-Siciliano et al., who reported sustained CAL gains of approximately 3 mm in EMD-treated sites after five years [3]. It is also in line with the findings of meta-analysis showing mean PPD reductions of 3.0–3.5 mm with PRF [12, 23]. Taken together, the results confirm that both materials can produce clinically meaningful improvements that are stable over extended periods.

Hard tissue changes, assessed by bone sounding, followed a similar trajectory in both groups. At six months, PRF-treated sites improved from 10.46 ± 2.63 mm to 6.38 ± 1.66 mm, while EMD-treated sites improved from 9.53 ± 2.54 mm to 5.92 ± 1.19 mm. At three years, however, BS values in both groups had slightly increased to 7.0 mm, suggesting some remodelling of regenerated tissues. Similar trends have been described in long-term clinical studies by Sculean et al. and Trombelli et al., where initial bone fill was followed by minor adjustments over time [6, 24]. These changes likely reflect normal physiological bone remodelling rather than a true relapse of disease, underscoring the importance of long-term follow-up when evaluating regenerative outcomes.

Overall, our results indicate that PRF and EMD achieve comparable long-term regenerative outcomes, although their clinical profiles differ in the early postoperative phase. PRF appears to confer a transient soft tissue advantage by limiting gingival recession, while EMD continues to be supported by extensive histological and clinical evidence as the most predictable material for achieving true periodontal regeneration, including the formation of new cementum, periodontal ligament, and alveolar bone [15, 25, 26]. From a clinical perspective, PRF represents a cost-effective and autologous alternative, especially in patients for whom minimizing postoperative recession is a priority or where the use of autologous biomaterials is preferred. Nevertheless, the wealth of histological data supporting EMD underscores its role as the current gold standard in periodontal regeneration.

Future research should aim to validate these findings in larger, multi-centre randomized trials with longer follow-ups and advanced three-dimensional imaging techniques such as CBCT. Such studies would help to determine whether the early soft tissue benefits of PRF can translate into distinct long-term advantages and clarify its role relative to EMD in regenerative periodontal therapy.

## Conclusions

Our findings suggest that the autologous PRF represents a viable and cost-effective alternative to EMD for periodontal healing. One noteworthy observation was that PRF demonstrated a short-term advantage in reducing postoperative gingival recession, however longer term outcomes were comparable between the two groups. Another limitation of the present study is the absence of quantitative radiographic assessment. Although representative radiographic images were obtained during follow-up, no standardized linear radiographic measurements or CBCT-based analyses were performed to evaluate hard tissue changes. Therefore, conclusions regarding radiographic bone fill should be interpreted with caution. Future studies with larger populations, human histology and radiographic assessments will further elucidate the regenerative potential of PRF.

## Data Availability

The datasets generated and/or analyzed during the current study are available from the corresponding author on reasonable request.

## Abbreviations

EMD: Enamel matrix derivate
PRF: Platelet rich fibrin
PPD: Probing pocket depth
GR: Gingival recession
CAL: Clinical attachment loss
BS: Bone sounding
p-ERK: Signal-regulated kinase and
OPG: Osteoprotegerin
ALP: Alkaline phosphatase
PDGF: Platelet derived growth factor
TGF-β: Transforming growth factor beta
IGFs: Insulin like growth factor
TNF-α: Tumor necrosis factor-alpha
IL-1β: Interleukin 1beta
IL-6: Interleukin 6
IL-4: Interleukin 4
VEGF: Vascular endothelial growth factor
PI: Plaque index
FMPS: Full mouth plaque score
GI: Gingival index
Mv: Mid-vestibular
V: Vestibular
Dv: Disto-vestibular
Mo: Mesio-oral
O: Oral
Do: Disto-oral
CEJ: Cemento-enamel junction
SD: Standard deviation
PPP: Platelet poor plasma
PRF: Platelet rich fibrin
RBC: Red blood cells
EDTA: Ethylenediaminetetraacetic
